# Assessment of COVID-19 Fear in Five European Countries before Mass Vaccination and Key Predictors among Nurses and Nursing Students

**DOI:** 10.3390/vaccines10010098

**Published:** 2022-01-10

**Authors:** Evridiki Patelarou, Petros Galanis, Enkeleint A. Mechili, Agathi Argyriadi, Alexandros Argyriadis, Evanthia Asimakopoulou, Emirjona Kicaj, Jorgjia Bucaj, Juan Manuel Carmona-Torres, Ana Isabel Cobo-Cuenca, Jakub Doležel, Stefano Finotto, Darja Jarošová, Athina Kalokairinou, Daniela Mecugni, Velide Pulomenaj, Krenar Malaj, Idriz Sopjani, Majlinda Zahaj, Athina Patelarou

**Affiliations:** 1 Department of Nursing, School of Health Sciences, Hellenic Mediterranean University, 714 10 Crete, Greece; apatelarou@hmu.gr; 2 Clinical Epidemiology Laboratory, Department of Nursing, National and Kapodistrian University of Athens, 157 72 Athens, Greece; pegalan@nurs.uoa.gr (P.G.); athkal@nurs.uoa.gr (A.K.); 3 Clinic of Social and Family Medicine, School of Medicine, University of Crete, 700 13 Crete, Greece; mechili@univlora.edu.al; 4 Department of Healthcare, Faculty of Health, University of Vlora, 9401 Vlora, Albania; ekicaj@yahoo.it (E.K.); jorgjia.bucaj@univlora.edu.al (J.B.); majlinda.zahaj@univlora.edu.al (M.Z.); 5 Department of Psychology and Social Sciences, Frederick University, Nicosia 3080, Cyprus; pre.aa@frederick.ac.cy; 6 Department of Nursing, Frederick University, Nicosia 3080, Cyprus; hsc.arg@frederick.ac.cy (A.A.); hsc.ae@frederick.ac.cy (E.A.); 7 IMCU Group, Faculty of Physiotherapy and Nursing, University of Castilla-La Mancha, 45004 Toledo, Spain; JuanManuel.Carmona@uclm.es (J.M.C.-T.); AnaIsabel.cobo@uclm.es (A.I.C.-C.); 8 Department of Nursing and Midwifery, Faculty of Medicine, University of Ostrava, 701 03 Ostrava, Czech Republic; jakub.dolezel@osu.cz (J.D.); darja.jarosova@osu.cz (D.J.); 9 Department of Surgery, Medicine, Dentistry and Morphological Sciences, University of Modena and Reggio Emilia, 41121 Reggio Emilia, Italy; sfinotto@unimore.it (S.F.); daniela.mecugni@unimore.it (D.M.); 10 Degree Course in Nursing, Azienda USL-IRCCS di Reggio Emilia, 41121 Reggio Emilia, Italy; 11 Faculty of Nursing, AAB College, P.O. Box 10000 Pristina, Kosovo; velide.pulomemoj@universitetiaab.com (V.P.); idriz.sopjani@universitetiaab.com (I.S.); 12 Research Centre of Public Health, Faculty of Health, University of Vlora, 9401 Vlora, Albania; kmalaj3@yahoo.com

**Keywords:** nurses, nursing students, COVID-19, fear, FCV-19S

## Abstract

Background: Levels of fear have increased since the COVID-19 pandemic outbreak. The absence of a safe and effective vaccine for mass-vaccination deteriorates this situation, which has a significant impact on mental health. This study aimed to assess the feelings of fear among nurses and nursing students in five European countries. Methods: A multicenter cross-sectional study was conducted in five European countries (Greece, Albania, Cyprus, Spain, and Kosovo) before the start of mass vaccination in Europe. Data collection was conducted in December 2020–January 2021 using an online questionnaire for nursing students and professional nurses. Fear of COVID-19 Scale (FCV-19S) was used for measuring levels of fear. IBM SPSS version 21.0 was used for statistical analysis. Results: The study population included 1135 nurses and 1920 nursing students from Kosovo (*n* = 1085), Spain (*n* = 663), Greece (*n* = 534), Albania (*n* = 529), and Cyprus (*n* = 244). According to multivariable analysis, females (OR = 2.53, 95% CI = 1.89–3.15), married (OR = 0.86, 95% CI = 0.24–1.48), nurses (OR = 0.87, 95% CI = 0.28–1.45) and those with a chronic disease (OR = 0.86, 95% CI = 0.11–1.62) were more fearful of COVID-19. Conclusions: It is important to decrease fear in the population of nurses who are at the frontlines of the pandemic. The provision of appropriate education and training activities for nurses and students to manage their stress levels is of high importance. Future studies should focus on levels of fear after the administration of several safe and effective vaccines worldwide.

## 1. Introduction

Since the outbreak of the COVID-19 pandemic, there has been an increase in the level of fear of the virus in the general population and, consequently, an increase in scientific interest in studying this fear and its consequences on mental health. Ahorsu et al. [1] constructed a scale for measuring the public’s fear of COVID-19, and similar efforts have also been made in several studies using weighing scales to measure the fear of COVID-19 [2,3,4,5,6,7,8]. Research to date focuses on specific population groups in the United States [9], India [10], Pakistan [11], the Philippines [12], and Spain [4], but there is a significant research gap in studies involving southern Mediterranean countries, as well as multifactorial studies or other research efforts involving a large population sample in several countries simultaneously. What is certain, however, is the very existence of the population’s fear of the SARS-CoV-2 virus [10,13,14]. At the same time, insufficient data have been recorded for the population of professional nurses and nursing students, a population group that has come into extensive direct contact with COVID-19-positive cases. The first available research results have shown that the emergence of COVID-19 has significantly impacted the mental health and well-being of nurses [15,16,17], but even more relevant studies are needed so that the scientific community can come to safe conclusions. In the last two years, there has been significant research interest in the study of psychological readiness as well as in the psychological consequences experienced by health professionals, especially medical and nursing staff [18,19,20,21]. However, many health professionals with chronic conditions seem to suffer from higher levels of depression, stress, and anxiety [22]. The psychological pressure caused by this pandemic has been shown to lead to an increase in vaccinations for medical and nursing staff, as well as for nursing students [23,24]. The level of hesitation in terms of vaccinations has been researched, related to influenza vaccination, showing a positive correlation [25,26], as well as an increased interest from researchers in the existence of such a correlation. 

In fact, this discussion has highlighted issues of trust in the safety of vaccines for the protection of nurses and nursing students, as well as issues of trust in specialists and the governments that promote them [27,28,29]. There is still a lack of studies to compare the aforementioned trust and fear between professional nurses and nursing students; however, at this level, researchers have begun to work on this correlation [18] but have not reached generalized results.

From the above it becomes clear that the coronavirus has caused fear and unpleasant feelings in both the general population and among health professionals. To date, research on the experience of nurses and nursing students who first came across the coronavirus, from the entire social context in terms of feelings of fear, is quite limited, which aroused the interest of our research team and was the incentive for this study. This study aimed to assess feelings of fear among nurses and nursing students in five European countries.

## 2. Materials and Methods

A multicenter cross-sectional study was conducted in five countries (Greece, Albania, Cyprus, Spain, and Kosovo) during the so-called second wave of the COVID-19 pandemic. In brief, in all countries data was collected December 2020–January 2021. Our sample consisted of undergraduate nursing students who were attending online or face-to-face classes organized by 7 universities in participating countries, and professional nurses were also contacted via email (sent by relevant nurses’ associations and councils), newsletters, and social networks. Students and nurses were invited to participate in the study through a web survey, which included general information regarding the purpose and the process of the study. Before completing the questionnaire, participants gave their written consent to participate in the study. The questionnaire was anonymous and no personal data were recorded. Participation in the study was voluntary and individuals could withdraw at any moment. The questionnaire comprised 29 items, with a mean duration of 6–8 min to complete. Questions were included about demographic characteristics, perceived knowledge and beliefs regarding coronavirus and the COVID-19 vaccine, trust towards the experts, doctors, and government, and factors influencing the students’ intention to vaccinate against the COVID-19 virus. The second part of the instrument included the Fear of COVID-19 Scale (FCV-19S) which was used to measure the fear of the coronavirus [1]. Cronbach’s alpha coefficient for the fear of COVID-19 scale was 0.87 and thus internal consistency was great. Study details and questionnaire development and validation processes are provided elsewhere [24,30].

Continuous variables are presented as mean and standard deviation, while categorical variables are presented as numbers and percentages. We used mortality from COVID-19 (deaths per million population) to categorize the five countries in three categories: high mortality (>800 deaths per million population) [31], moderate mortality (400–800 deaths per million population), and low mortality (<400 deaths per million population). The low mortality group included Cyprus, the moderate mortality group included Greece, Kosovo, and Albania, and the high mortality group included Spain. Fear of COVID-19 was the dependent variable, while demographic data and participants’ answers regarding the COVID-19 pandemic and vaccination were the independent variables. Differences between nurses and nursing students were assessed with the chi-square test, chi-square trend test, and independent samples *t*-test. We created univariate and multivariable linear regression models. In the multivariable model, we included variables with a *p*-value < 0.20 in the univariate analysis, applying the backward stepwise model. In the regression models, we merged answers in some variables for theoretical and statistical reasons. In particular, we merged the answers “very low” and “low”, as well as the answers “high” and “very high”, since the meanings were the same. Moreover, in the item “accept a safe and effective COVID-19 vaccine” we considered the answers “somewhat agree” and “completely agree” as “yes” and the other answers as “no” since we wanted to make explicit the intention of participants to accept a COVID-19 vaccine or not. We estimated unadjusted and adjusted beta coefficients with 95% confidence intervals (CI) and *p*-values. All tests of statistical significance were two-tailed, and *p*-values less than 0.05 were considered significant. We used Statistical Package for Social Sciences software (IBM Corp. Released 2012. IBM SPSS Statistics for Windows, Version 21.0. IBM, Armonk, NY, USA) to perform statistical analyses.

## 3. Results

### Results and Data Analysis

Detailed demographic characteristics of the study population are presented in Table 1. The study population included 1135 nurses and 1920 nursing students; from Kosovo (*n* = 1085), Spain (*n* = 663), Greece (*n* = 534), Albania (*n* = 529), and Cyprus (*n* = 244). Nurses were older than nursing students (38.3 years vs. 21), and chronic disease was more frequent among nurses (21.1% vs. 5.8%). 

Study population answers regarding the COVID-19 pandemic are shown in Table 2. Nurses had come into contact with a confirmed or suspected case of COVID-19 more often than nursing students (81.5% vs. 49.6%, *p* < 0.001). Significant percentages of nurses and nursing students had been infected with COVID-19 (19.2% and 11.7%, respectively, *p* < 0.001). COVID-19 infections were even higher among participants’ family members/friends. In particular, 67.7% of nurses’ family members/friends had been infected with COVID-19, while the respective percentage for nursing students was 60.1% (*p* < 0.001). Self-perceived knowledge about COVID-19 was high/very high for both groups (65.1% for nurses and 60.2% for nursing students) but low/very low regarding COVID-19 vaccines (44.1% for nurses and 55.2% for nursing students). A significantly higher percentage of nurses was vaccinated for influenza in 2019 and 2020 (36.2% vs. 4.5%, *p* < 0.001). Trust in the government regarding information about COVID-19 was low (36.7%), but trust in doctors and government experts was moderate to high (72.2% and 63.1%, respectively). Willingness to accept a safe and effective COVID-19 vaccine was higher among nurses (*p* < 0.001). In particular, 65.3% of nurses somewhat/completely agreed to accept a novel COVID-19 vaccine, while the respective percentage for nursing students was 43.7%.

Nurses experienced more fear of COVID-19 than nursing students (mean score; 16.2 and 14.6, respectively, *p* < 0.001); the mean total score was 15.2 (standard deviation; 5.9) with a range from 7 to 35.

Results from univariate and multivariable linear regression analyses are shown in Table 3. According to multivariable linear regression model, females (OR = 2.53, 95% CI = 1.89–3.15) and married (OR = 0.86, 95% CI = 0.24–1.48) experienced more fear of COVID-19. Levels of COVID-19 fear were higher among nurses (OR = 0.87, 95% CI = 0.28–1.45) and those with a chronic disease (OR = 0.86, 95% CI = 0.11–1.62). Participants living in countries with higher mortality from COVID-19 were more afraid of the disease (middle vs. low mortality group; OR = 1.88, 95% CI = 1.02–2.75, high vs. low mortality group; OR = 2.82, 95% CI = 1.89–3.74).

## 4. Discussion

This study aimed to assess factors that affect fear of COVID-19 among nurses and nursing students in five countries during the ongoing pandemic. Nurses experienced more fear than nursing students. 

Similar results to our study were reported by Huang et al. [18]; the authors reported higher levels of fear among nurses than nursing college students. In general, nurses have been on the frontline throughout the pandemic. Their working environment brings them in daily contact with COVID-19 patients, and exposes them more to the disease. Fear of being infected, the possible infection of family members, infected colleagues and friends, daily contact with COVID-19 patients who died from the disease, long working hours, and the unknowns of the disease are the most probable key reasons for these results. Additionally, being afraid of infecting relatives or friends increases the levels of fear and stress among nurses, as reported by Huang et al. [18]. A study in the Philippines reported high levels of fear due to COVID-19 among nurses [32], while a study in China among frontline nurses reported moderate and high levels of fear [33]. Another study found that nursing students exhibit less fear and sadness than frontline nurses [18]. Psychological distress during patient care and patient loss despite nurses’ efforts to save them are other possible reasons for these results [18,34]. However, a lack of participation in COVID-19 training activities and job insecurity (working part-time) are other important factors, as mentioned in another study [32]. Level of health literacy is directly connected with COVID-19 fear [35]; however, this contrasts with the current study. Nurses in frontline positions have more knowledge and are probably more afraid because they know the severity of the disease, while nursing students are younger and probably overestimate their health status. Additionally, lack of medical equipment (especially during the first period of the pandemic) and fear of infection and possible isolation could also explain the higher levels of fear among nurses. However, further studies are needed to confirm this.

In the current study, we saw that women are more likely to present higher level of stress than men. High levels of anxiety are reported in studies among nursing students and frontline nurses [33,36]. Additionally, women have reported higher level of fear in comparison to men in different studies [33,35,37]. The female gender tends to relate more with anxiety and psychological distress [38]; to some extent, a Cuban study concluded that female gender is a key predictor of medium and high level of fear of COVID-19 [37]. The results of the present study could be due to the much higher number of women who participated in the study in comparison to men. However, similar results have been reported to general population studies suggesting that women are experiencing the COVID-19 situation worse than men [2,39,40]. The prevalence of mood and anxiety disorders are usually higher in women than men [41]. These results more likely relate to biological, social, and psychosocial factors. Women’s role in society is complex. They are involved in all aspects of life, participating in both housekeeping and the job market. These increased responsibilities activate stress mechanisms. In contrast, men are usually more indifferent to various aspects of life and experience different stressful situations more easily. However, female nurses reported a higher level of insomnia in a study in Wuhan than men [41]. Having sleep problems may also increase levels of fear.

Participants from countries with middle and high mortality rates due to COVID-19 reported higher levels of stress in this research. A study among nursing students reported higher levels of depression for Spanish students than Albanians and Greeks [42]. Spain death rates per 100,000 people for COVID-19 are much higher than Greece and Albania [31]. Those who follow the daily numbers of infection and death from COVID-19 experience more fear. Fear of infecting themselves and possible infection of their loved ones that could lead to serious problems and/or death could explain these results. The high volume of information received from traditional and non-traditional media could also impact these results. The ongoing “infodemic” during this period could be an additional factor, especially in the most affected countries.

The present study reports that marital status was also connected with levels of fear. Married participants reported higher fear levels than single/widowed/divorced participants. Hendrick and Hendrick reported that “love in the age of the Coronavirus is challenging” [43]. Most of the nurses participating in the current study are older and married in comparison to nursing students. Being afraid of getting infected and a possible transmission to their family members increase the levels of fear. Additionally, as nurses are also often the breadwinner for their families, possible financial hardships due to the COVID-19 situation could also be an explanatory factor. In contrast to the results of the current work, a study among primary medical staff in China reported that being married was a protective factor for development of psychological problems [26]. Support from the family environment is a possible factor for decreasing stress levels. Additionally, an Israeli study reported that martial satisfaction of women is linked with fear of COVID-19 [44]. This factor was not assessed in this study and future research is needed.

Participants with a chronic condition reported higher levels of stress in comparison to those without. Similar results are reported by the study of Mekonen et al. [22]; the authors concluded that levels of depression, stress, and anxiety are much higher for nurses with chronic conditions in comparison to those who do not. In a study of students, having a chronic medical condition was associated with high risk of stress [45]. Another study among frontline nurses in China reported that insomnia is related to existence of chronic conditions [46]. People with a chronic condition are more vulnerable to an infection with COVID-19. A possible infection in synergy with the existing health problem can worsen the situation. This combination probably stresses participants more and causes fear. However, a study conducted in Turkey reported that people without a chronic condition are more likely to have high anxiety levels in comparison to those with a chronic problem. Those authors believe that those with a medical condition are more aware of the disease and take better care of themselves [47]. Differences in education level of participants and years living with the chronic problem may be key reasons for these variations. Acceptance of a safe and effective COVID-19 vaccine was different between nursing students and nurses in the current study. In general, nurses were of higher age and at higher risk of serious illness and/or death in comparison to students who were younger. Additionally, working in hospitals puts nurses on the frontline, which also makes them vulnerable and this probably increased the acceptance rate. In a study among Italian university students, COVID-19 vaccine hesitance rates ranged between 22–29% [48]. Additionally, authors reported that healthcare students had higher COVID-19 vaccine acceptance rates in comparison to other students [48]. In a study from the USA among medical students, around 25% of them were hesitant of having the COVID-19 vaccine [49]. The authors suggested the development of an educational curriculum for increasing knowledge and decreasing hesitancy levels [49]. A total of 88.3% of nursing students in a study in the Eastern USA believe that COVID-19 vaccines are safe and the acceptance rate was at 92% [50].

## 5. Strengths and Limitations

To our best knowledge, this is among the first studies that aimed to assess factors that affect fear of COVID-19 among nurses and nursing students in five European countries. Large geographical coverage and an extensive number of participants are among the key strengths of the current research. Results also indicate key factors that could be addressed to decrease fear level of nurses and nursing students.

As with all studies, this work suffers from some limitations. The cross-sectional nature of the research makes it difficult to draw conclusions about causalities. Additionally, there was a lack of representativeness in each country during data collection. Participation of only nurses and nursing students makes generalization for other medical professions difficult. The online method of data collection is also a possible limitation for this study.

## 6. Conclusions

The current study focused on factors that affect levels of fear among nurses and nursing students before mass vaccination started in Europe. Key predictors of high levels of fear were female gender, marital status, existence of a chronic medical condition, and living in a country with high mortality rates due to COVID-19. Nurses are in the frontline of the pandemic while nursing students are the forthcoming health providers. It is of paramount significance to decrease the level of fear in these populations. Provision of appropriate education for students and training activities for nurses to manage their stress levels is of high importance. Researchers, policymakers, health authorities, and nurses’ associations should work together to find appropriate and suitable coping strategies. Future research is needed to assess if differences exist between nurses and nursing students and other medical professions. Additionally, further studies should focus on the levels of fear after vaccination and assess if this process contributes to a better health status.

## Figures and Tables

**Table 1:** Demographic characteristics of the study population.

Characteristics	Nursing Students		Nurses		*p*-Value
	*n*	%	*n*	%	
Gender					0.6 ^a^
Male	307	16.0	173	15.3	
Female	1610	84.0	960	84.7	
Age (years), mean, standard deviation	21.0	4.6	38.3	10.9	<0.001 ^b^
Country of origin					<0.001 ^a^
Albania	313	16.3	216	19.0	
Greece	275	14.3	259	22.8	
Spain	181	9.4	482	42.5	
Cyprus	131	6.8	113	10.0	
Kosovo	1020	53.1	65	5.7	
Marital status					<0.001 ^a^
Single	1735	91.1	445	39.3	
Married	153	8.0	601	53.1	
Widowed	2	0.1	8	0.7	
Divorced	15	0.8	77	6.8	
Chronic disease					<0.001 ^a^
Yes	111	5.8	240	21.1	
No	1806	94.2	895	78.9	
Living with vulnerable groups during the COVID-19 pandemic					<0.001 ^a^
Yes	785	40.9	384	33.8	
No	1133	59.0	751	66.2	

^a^ chi-square test; ^b^ independent samples *t*-test.

**Table 2:** Study population answers regarding COVID-19.

	Nursing Students		Nurses		*p*-Value
	*n*	%	*n*	%	
Contact with a confirmed or a suspected case of COVID-19					<0.001 ^a^
Yes	951	49.6	924	81.5	
No	965	50.4	210	18.5	
Infected with COVID-19					<0.001 ^a^
Yes	225	11.7	218	19.2	
No	1693	88.3	915	80.8	
Family members/friends infected with COVID-19					<0.001 ^a^
Yes	1150	60.1	766	67.7	
No	764	39.9	366	32.3	
Self-perceived likelihood of getting infected with COVID-19 in the future					<0.001 ^b^
Very low	69	3.9	20	2.2	
Low	410	22.9	110	12.0	
Moderate	862	48.1	348	38.0	
High	326	18.2	314	34.3	
Very high	124	6.9	124	13.5	
Self-perceived knowledge about COVID-19					<0.001 ^b^
Very low	16	0.8	2	0.2	
Low	55	2.9	25	2.2	
Moderate	692	36.1	369	32.6	
High	918	47.8	552	48.8	
Very high	238	12.4	184	16.3	
Self-perceived knowledge about COVID-19 vaccines					<0.001 ^b^
Very low	361	18.8	133	11.7	
Low	699	36.4	367	32.4	
Moderate	680	35.4	432	38.1	
High	132	6.9	153	13.5	
Very high	48	2.5	48	4.2	
Influenza vaccination in 2019 and 2020					<0.001 ^a^
No	1830	95.5	724	63.8	
Yes	86	4.5	411	36.2	
Trust in government					0.1 ^a^
Yes	680	35.6	436	38.6	
No	1230	64.4	694	61.4	
Trust in doctors regarding information about COVID-19					0.1 ^a^
Yes	1363	71.1	836	74.0	
No	553	28.9	294	26.0	
Trust in government experts regarding information about COVID-19					0.7 ^a^
Yes	1214	63.3	708	62.6	
No	703	36.7	423	37.4	
Accept a safe and effective COVID-19 vaccine					<0.001 ^b^
Completely disagree	233	12.1	55	4.8	
Somewhat disagree	205	10.7	43	3.8	
Neutral	642	33.4	296	26.1	
Somewhat agree	465	24.2	334	29.4	
Completely agree	375	19.5	407	35.9	

^a^ chi-square test; ^b^ chi-square trend test.

**Table 3:** Univariate and multivariable linear regression analysis with fear against COVID-19 as the dependent variable.

Variable	Unadjusted Coefficient Beta (95% CI)	*p*-Value	Adjusted Coefficient Beta (95% CI)	*p*-Value
Gender (females vs. males)	2.82 (2.24 to 3.39)	<0.001	2.53 (1.89 to 3.15)	<0.001
Age	0.07 (0.05 to 0.09)	<0.001	NS	
Nurses vs. nursing students	1.61 (1.18 to 2.05)	<0.001	0.87 (0.28 to 1.45)	0.004
Mortality group per million population				
Low	1.00 (reference)		1.00 (reference)	
Middle	−0.60 (−1.05 to −0.16)	0.007	1.88 (1.02 to 2.75)	<0.001
High	1.41 (0.94 to 1.88)	<0.001	2.82 (1.89 to 3.74)	<0.001
Marital status (married vs. single/widowed/divorced)	1.71 (1.22 to 2.20)	<0.001	0.86 (0.24 to 1.45)	0.007
Chronic disease (yes vs. no)	1.73 (1.06 to 2.39)	<0.001	0.86 (0.11 to 1.61)	0.024
Living with vulnerable groups during the COVID-19 pandemic (yes vs. no)	0.007 (−0.005 to 0.018)	0.27	NS	
Contact with a confirmed or a suspected case of COVID-19 (yes vs. no)	0.62 (0.19 to 1.06)	0.005	NS	
Infected with COVID-19 (yes vs. no)	0.09 (−0.51 to 0.70)	0.75	NS	
Family members/friends infected with COVID-19 (yes vs. no)	0.15 (−0.29 to 0.59)	0.50	NS	
Self-perceived likelihood of getting infected with COVID-19 in the future				
Low/very low	1.00 (reference)			
Moderate	0.32 (0.14 to 0.78)	0.17	NS	
High/very high	0.71 (0.23 to 1.19)	0.004	NS	
Self-perceived knowledge about COVID-19				
Low/very low	1.00 (reference)			
Moderate	0.08 (−0.36 to 0.52)	0.718	NS	
High/very high	0.09 (−0.35 to 0.53)	0.70	NS	
Self-perceived knowledge about COVID-19 vaccines				
Low/very low	1.00 (reference)			
Moderate	−0.01 (−0.45 to 0.43)	0.96	NS	
High/very high	−0.14 (−0.78 to 0.51)	0.68	NS	
Influenza vaccination in 2019 and 2020 (yes vs. no)	0.87 (0.59 to 1.15)	<0.001	NS	
Trust in government (yes vs. no)	0.29 (−0.16 to 0.73)	0.203	NS	
Trust in doctors regarding information about COVID-19 (yes vs. no)	0.58 (0.11 to 1.05)	0.017	NS	
Trust in government experts regarding information about COVID-19 (yes vs. no)	0.12 (−0.32 to 0.56)	0.586	NS	
Accept a safe and effective COVID-19 vaccine (yes vs. no)	0.88 (0.46 to 1.31)	<0.001	NS	

CI: confidence interval; NS: not selected by the backward elimination procedure in the multivariable linear regression analysis with a significance level set at 0.05 and the R2 for the final multivariable model was 5.7%.

## Data Availability

Data can be found upon request from the corresponding author.
